# Engineering hydrogen bonding to align molecular dipoles in organic solids for efficient second harmonic generation†

**DOI:** 10.1039/d2sc03994j

**Published:** 2022-10-11

**Authors:** Ruyan Zhao, Tong Zhu, Sasa Wang, Charlie Jarrett-Wilkins, Amin Morteza Najjarian, Alan J. Lough, Sjoerd Hoogland, Edward H. Sargent, Dwight S. Seferos

**Affiliations:** Department of Chemistry, University of Toronto 80 St. George Street Toronto Ontario M5S 3H6 Canada dwight.seferos@utoronto.ca; Department of Electronic and Computer Engineering, University of Toronto 10 King's College Road Toronto Ontario M5S 3G4 Canada ted.sargent@utoronto.ca; Department of Chemical Engineering and Applied Chemistry, University of Toronto 200 College Street Toronto Ontario M5S 3E5 Canada

## Abstract

Considering nearly infinite design possibilities, organic second harmonic generation (SHG) molecules are believed to have long-term promise. However, because of the tendency to form dipole-antiparallel crystals that lead to zero macroscopic polarization, it is difficult to design a nonlinear optical (NLO) material based on organic molecules. In this manuscript, we report a new molecule motif that can form asymmetric organic solids by controlling the degree of hydrogen bonding through protonation. A conjugated polar organic molecule was prepared with a triple bond connecting an electron-withdrawing pyridine ring and an electron-donating thiophene ring. By controlling the degree of hydrogen bonding through protonation, two different crystal packing motifs are achieved. One crystallizes into the common dipole-antiparallel nonpolar *P*1̄ space group. The second crystallizes into the uncommon dipole-parallel polar *P*1 space group, in which the molecular dipoles are aligned along a single axis and thus exhibit a high macroscopic polarization in its solid-state form. Due to the *P*1 polar packing, the sample can generate second harmonic light efficiently, about three times the intensity of the benchmark potassium dihydrogen phosphate. Our findings show that crystal engineering by hydrogen bonding in a single molecular backbone can be used for controlling the macroscopic NLO properties.

## Abstract 2

We switch the molecule from SHG inert to SHG active by controlling the degree of hydrogen bonding through protonation.

## Graphical abstract

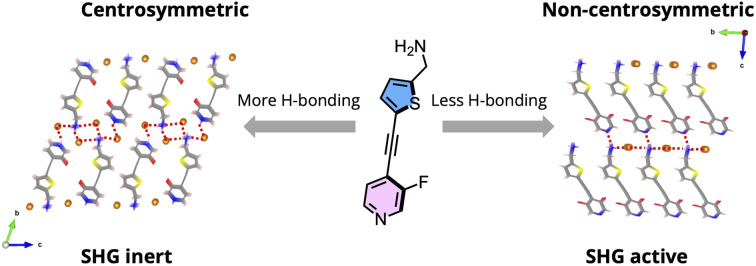

## Introduction

Second harmonic generation (SHG) active materials are non-centrosymmetric, and play an indispensable role in modern laser-related sciences and technologies because they enable nonlinear optical (NLO) processes that lead to the conversion of an optical input wave into an output wave with twice the input frequency.^1,2^ Compared to the inorganic SHG crystals, such as LiNbO_3_, AgGaS_2_, GaP, ZnTe, and GaAs, organic molecules have more long-term promise due to their high second-order nonlinear optical coefficients, ultrafast response times, and nearly infinite design possibilities.^3–8^

Efficient SHG organic molecules are usually designed based on π-conjugated systems, due to their high polarizability and delocalized electronic structure.^9–12^ Among them, π-conjugated molecules with electron-rich rings and electron-deficient rings connected through conjugated bridges can yield large molecular dipole moments that induce high hyperpolarizability (*β*), which is an important figure-of-merit related to the magnitude of the second-order nonlinear susceptibility (*χ*^2^). Unfortunately, these molecules tend to exhibit zero macroscopic polarization in the solid-state because they tend to form energetically favourable dipole-antiparallel assemblies by dipole–dipole interactions. Thus, it is a great challenge to design an NLO-active organic molecule that exhibits a large nonlinearity on the molecular level as well as on the macroscopic level.^13–15^

Crystal engineering through inductive intermolecular interactions to stabilize the dipole-parallel packing paves the way towards achieving a non-centrosymmetric molecular assembly. Several methods have been utilized to stabilize the dipole-parallel molecular alignment, such as using asymmetric porous host structures to trap molecules inside the channels,^16^ utilizing Langmuir–Blodgett (LB) film with asymmetry surfaces,^17^ and applying an external field to pole the material from symmetric to asymmetric.^18^ Ionic crystals consisting of molecular cations and anions have also been used to create stable non-centrosymmetric crystals utilizing the formation of strong electrostatic coulombic interactions and various π–π interactions between cation and anion layers.^2^ However, the introduction of multiple intermolecular and intramolecular interactions will increase the molecular and synthetic complexity towards achieving the targeted ordering in the crystalline state.

**Fig. 1:**
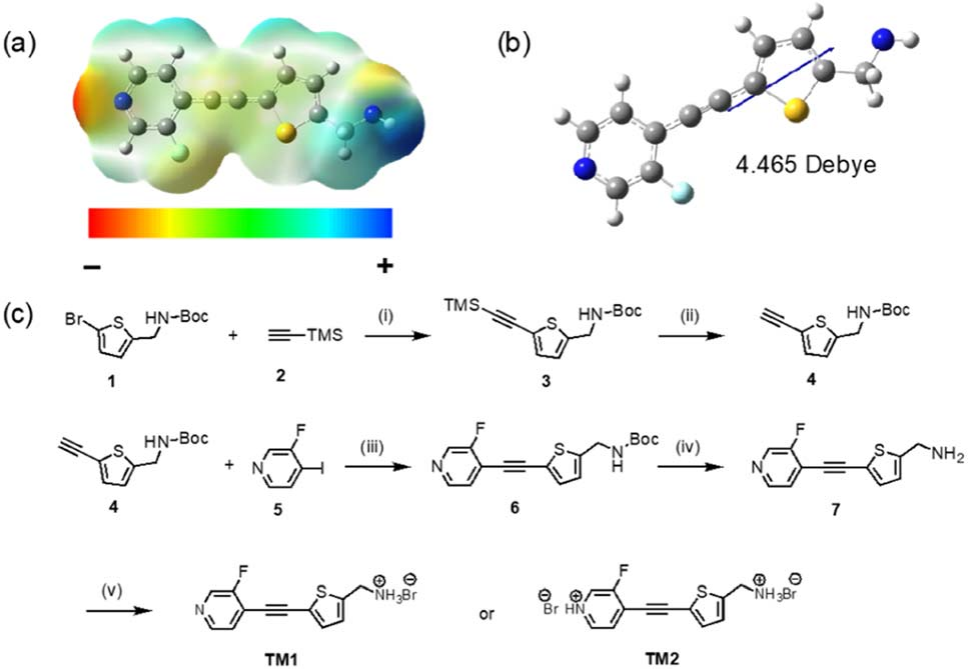
(a) Electrostatic potential (ESPs) map of neutral TM1. (b) Illustration of the molecular dipole moment of neutral TM1 (basis set: b3lyp/6-311G(d,p)). (c) Synthesis of TM1 and TM2. (i) Pd(PPh_3_)_2_Cl_2_, CuI, DIPA, THF, r.t., 12 h, 90%; (ii) TBAF in THF (1 M), THF : H_2_O = 5 : 1, r.t., 1 h, 98%; (iii) Pd(PPh_3_)_2_Cl_2_, CuI, DIPA, THF, r.t., 12 h, 83%; (iv) trifluoroacetic acid, DCM, r.t., 1.5 h, 86%; (v) 1 M HBr, methanol, 0 °C, 1.5 h, 92%.

In this work, we designed and synthesized a polar organic molecule, which exhibit macroscopic polarization by controlling the degree of hydrogen bonding between the molecules. Our molecule contains a large dipole from an electron-deficient pyridine ring and electron-rich thiophene ring connected by a carbon–carbon triple bond. The hydrogen bonding acceptor nitrogen atom in the pyridine ring and the hydrogen bonding donor nitrogen atom in the amine group are deliberately installed at the two ends of the molecule (Fig. 1a). By enlarging the charge separation distance through the triple bond, a large molecular dipole of 4.465 Debye can be realized (Fig. 1b). Significantly, different crystal packing motifs are observed when controlling the hydrogen bonding numbers and strength. Specifically, the version that only forms hydrogen bonds between the nitrogen atom in the amine group and the Br ion adopts a *P*1 non-centrosymmetric packing in the solid-state and exhibits a large SHG response. Further protonation leads to hydrogen bonds on both nitrogen atoms and pyridine, resulting in a packing order with inversion of symmetry that does not exhibit SHG.

## Results and discussion

### Molecular design

The electron-density distribution of the neutral molecule was simulated by plotting its electrostatic potential (ESPs) map (Fig. 1a). The end nitrogen in the pyridine ring has the strongest electron-withdrawing ability that is rendered in “red”. Meanwhile, the end nitrogen in the amine group has the strongest electron-donating ability that is rendered in “blue”. Because of the separation of the positive and negative charge center, the molecule exhibits a large dipole moment. HOMO and LUMO orbitals delocalized well on the whole backbone due to the good planarity of the molecule (Fig. S5†).

### Molecular synthesis

TM1 was synthesized with an overall yield of 57% through a 5-step synthesis process (Fig. 1c). The core framework was furnished by Sonogashira coupling between *tert*-butyl((5-ethynylthiophene-2-yl)methyl)carbamate 4 and 3-fluoro-4-iodopyridine 5. After purification, the –NH_2_ group was recovered by cleaving the –Boc group, followed by protonation using dilute HBr acid to produce the final salt, TM1. Adding more HBr will lead to further protonation of pyridine, which can be observed by an obvious color change from white (TM1) to yellow (TM2). The structure of TM1 and TM2 was characterized using pXRD, NMR (^1^H, ^13^C), and Fourier transform infrared (FTIR) measurements. Compared with the neutral compound 7, the chemical shift of the protons of –CH_2_ adjacent to the end nitrogen atom in TM1 and TM2 downfield, from 4.07 ppm to 4.31 ppm. The protons of residue water in DMSO are also shifted downfield (3.3 ppm in TM1 to 3.7 ppm in TM2) due to protonation to H_3_O^+^ by the residual free acid in TM2.

### Structure characterization

**Fig. 2:**
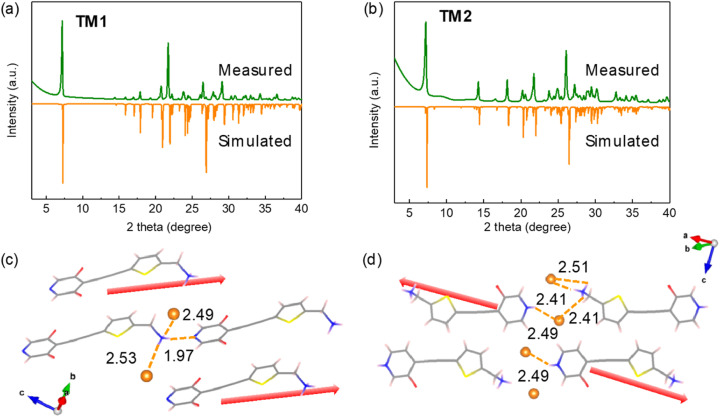
(a and b) pXRD patterns of as-synthesized powder of TM1 and TM2 and simulated from the CIF file. Crystal packing structure and the illustration of the supramolecular interactions of (c) TM1 and (d) TM2. Yellow dashed lines indicate the hydrogen bonding and the numbers indicate the distance in unit of Å. Red arrows indicate the direction of molecular dipoles.

Powder X-ray diffraction (pXRD) patterns of the as-synthesized powder samples of TM1 and TM2 are in good accordance with the simulated ones, suggesting the phase purity of the as-synthesized powder samples (Fig. 2a and b). FTIR was utilized to further characterize the presence of various functional groups in the as-synthesized powder of TM1 and TM2 (Fig. S1†). The stretching vibration of N–H in –NH_3_^+^ group becomes broad and moves to lower wavenumbers (3000 cm^−1^) compared with reported non-protonated –NH_2_ (>3400 cm^−1^), making those vibrations indistinguishable and overlapping with the stretching vibration of C–H bond at both pyridine and thiophene rings. The strong and sharp peak at 2206 cm^−1^ refers to the stretching vibration of C≡C. The asymmetric and symmetric bending vibration absorbing peaks of N–H in –NH_3_^+^ groups in TM1 are observed at 1608 cm^−1^ and 1494 cm^−1^, respectively. The stretching vibrational frequency peaks of non-saturated double bonds (C=N, C=C) are also observable in the range of 1610–1500 cm^−1^. Peaks in the figure print region (1350–400 cm^−1^) correspond to both in-plane (1320–1027 cm^−1^) and out-of-plane (817 cm^−1^) bending vibrations of unsaturated C–H and the stretching vibration of C–F. Hydrogen bonding is much stronger in TM2 than in TM1, thus the stretching vibration of N–H in –NH_3_^+^ group moves to low wavenumbers with increased intensity and band broadening. Meanwhile, the peaks of bending vibration of N–H in TM2 move to high wavelength numbers to 1634 cm^−1^ (asymmetric bending) and 1508 cm^−1^ (symmetric bending).^19,20^ Hydrogen bonding plays an important role in determining the final crystal stacking, where suitable hydrogen bonding numbers and strength will lead to a stable energetically-unfavourable polar packing.

Single crystals of TM1 and TM2 were obtained by the anti-solvent method. TM1 adopts a triclinic crystal system with a non-centrosymmetric space group of *P*1 (Fig. 2c), in which molecular dipoles are aligned in one direction. TM1 and TM2 crystals show a similar short π–π stacking distance of *ca.* 3.4 Å, indicating the π–π interactions do not account for the packing transformation (Fig. S4†). Multiple supramolecular interactions, present in TM1, are essential to stabilize the dipole-parallel configuration. Moreover, the three hydrogen atoms on the end amine group form a hydrogen bonding network with the neighboring bromine and nitrogen atoms. Especially the formation of hydrogen bonds between the N1 (in amine group) and N (in pyridine ring) helps to create a dipole-parallel configuration that induces a macroscopic dipole. The molecular ordering changes when more hydrogen bonding is introduced by adding more HBr acid to protonate pyridine to pyridinium. The crystals of TM2 exhibit inversion symmetry with a non-polar space group of *P*1̄ (Fig. 2d). In TM2 crystals, the N–H group of the pyridinium cannot form hydrogen bonding with N1 (in amine group)–H group, and instead forms hydrogen bonding with the surrounding Br, thus transforming the polar stacking to nonpolar stacking.

### Nonlinear properties

**Fig. 3:**
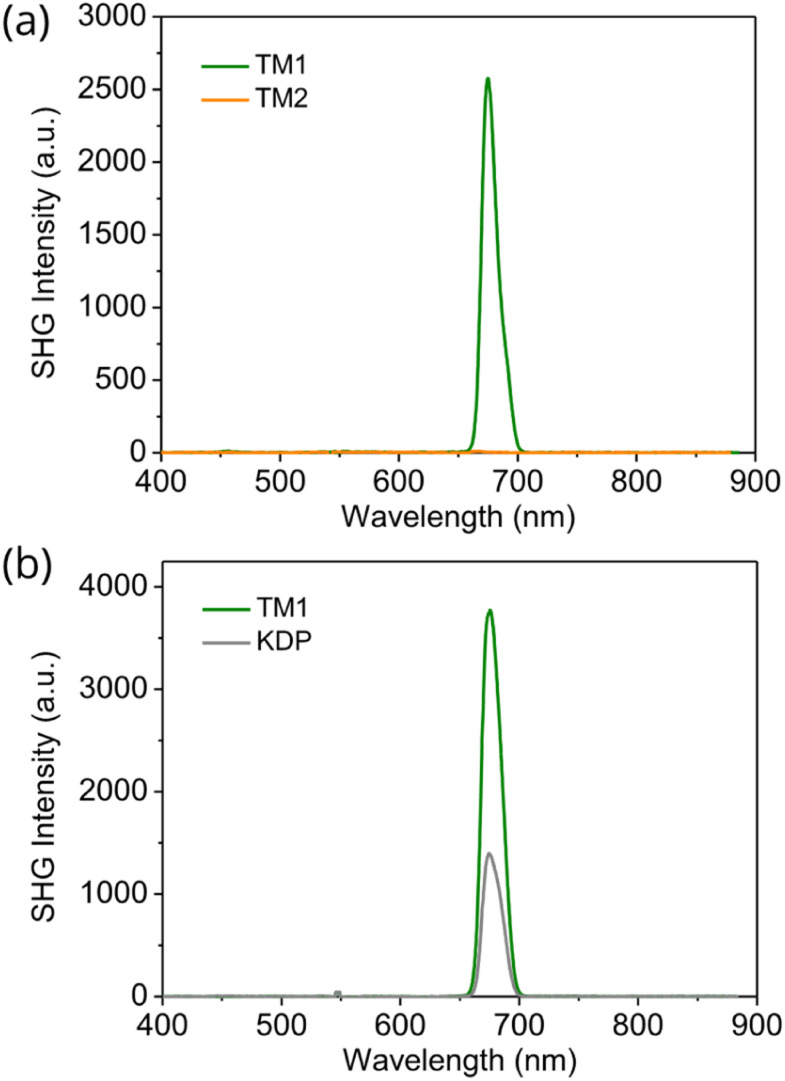
(a) SHG signal from TM1 and TM2 single crystal powder. (b) SHG intensity of TM1 and KDP at the same particle size of 100–150 μm at 1350 nm.

SHG measurements further confirm the different crystal packing of TM1 and TM2. SHG measurements were performed by directing 1350 nm incident laser light to the powder samples that were created by grinding the single crystals. Because of lacking inversion symmetry, TM1 exhibits an SHG response at the wavelength of 675 nm which is half the input wavelength. Meanwhile, TM2 shows a negligible SHG signal (Fig. 3a). Moreover, the SHG intensity of TM1 increases with increasing average particle size under 200 μm, and then tends to remain unchanged when the particle size is over 200 μm, indicating its good phase matching ability based on the Kurtz–Perry method.^21,22^ To gain further insight into the SHG intensity of TM1, we compared it with potassium dihydrogen phosphate (KDP) crystal, a standard inorganic SHG crystal, with the same particle size (about 100–150 μm). We found that the SHG intensity of TM1 is about three times higher compared to that of KDP (Fig. 3b), making our SHG active organic molecules among the best reported.^5,9,10,23–26^

Optical transparency and stability are two critically important properties for the application of any NLO material. Considering its optical transparency and high thermal stability, TM1 is among the best SHG active molecules when compared with other representative ones (Fig. S8a, b and Table S1†). The optical transparency of the TM1 was investigated by measuring UV-vis spectra in transmittance mode (Fig. S2†). The sample was prepared by sandwiching TM1 powder between two glass slides. TM1 is transparent to the light with a wavelength higher than 364 nm. According to the onset absorption wavelength (347 nm), the bandgap of TM1 was calculated to be 3.57 eV, which agrees well with the simulated bandgap of 3.07 eV (Fig. S9†). Thermogravimetry analysis (TGA) was carried out from 30 °C to 800 °C at a heating speed of 10 K min^−1^ under the protection of nitrogen (Fig. S3†). TM1 is stable up to 243 °C. After heating to 800 °C, 51% of the original mass remained in the crucible, corresponding to the carbon content in TM1.

The single crystal XRD analysis was insufficient to resolve the positions of the fluorine atoms as they are not fixed in a single position. There is a small amount of disorder of the F atom about the two ortho sites. As shown in the potential scan plots (Fig. S6†), the energy difference between the two configurations is 0.97 kJ mol^−1^, which is smaller than the thermal energy at room temperature (*kT* = 2.51 kJ mol^−1^). The position of the fluorine atom has an influence on the overall stacking of the molecules inside the crystal, and correspondingly will influence the macroscopic polarization.

### DFT simulation

**Fig. 4:**
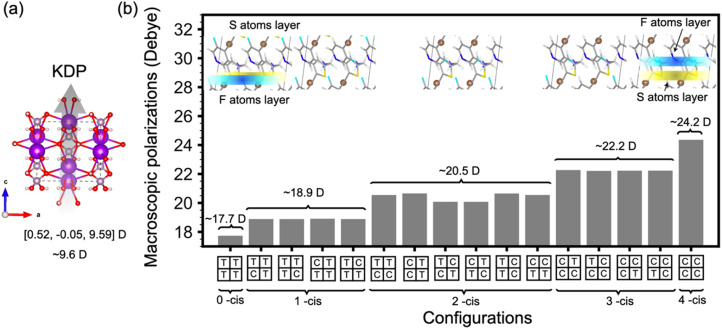
DFT calculated relative macroscopic polarization for (a) KDP and (b) all 16 possible alignments of TM1 crystals based on a 2 × 2 × 1 supercell. All the sixteen possible alignments are separated into five different groups according to the number of -*cis* conformations (*C* for -*cis* conformations and *T* for -*trans* conformations) within the absolute calculated Berry phase polarizations values labelled on top of them. For each group, one represented crystal structure is shown, and the average S atoms layer is represented in yellow and the average F atoms layer is represented in cyan.

We used Density Function Theory (DFT) simulations to elucidate this phenomenon. We used a 2 × 2 × 1 supercell to explore the position effects of fluorine atoms on the macroscopic packing and polarizations. All the sixteen possible alignments of TM1 crystals (Fig. S9†) are considered in this work. Compared to the very small deviation of the total energies (largest difference around 0.046 eV per formula) from different alignments, the bandgap/Berry phase polarization shows a large deviation around 0.15 eV/6.62 Debye, within a linear dependence of the -*cis* and -*trans* conformations of the units in the supercell (Fig. S9†). The smallest polarization of TM1 crystals is 17.7 Debye, which is about twice that of KDP (9.6 D). We found that with more -*cis* conformations in the supercell, the macroscopic polarization increases and the bandgap decreases. Furthermore, by looking through the detailed crystal packing (Fig. 4), we found that the large increase of the macroscopic polarization from more -*cis* conformations is a result of the successful separation of the layered packing of fluorine atoms and sulfur atoms.

## Conclusions

In conclusion, we have synthesized an organic molecule TM1 with a large molecular dipole moment by connecting a highly electron-withdrawing pyridine ring and the strongly electron-donating thiophene ring through a triple bond. Hydrogen bonding allows the molecule to align molecular dipoles in a single direction resulting in NLO activity. As a result, this material exhibits a strong second harmonic generation signal, about three times the intensity of the benchmark KDP, showing that this material is amongst the best of SHG organic molecules. This is in contrast to the crystals made with the control molecule TM2, which exhibited an inversion symmetry and correspondingly no second harmonic generation signal. Our findings not only provide a useful NLO material with efficient SHG response, high optical transparency, and excellent thermal stability, but also shed light on crystal engineering using hydrogen bonding to obtain NLO materials. The results are expected to be general to other molecules that undergo hydrogen bonding and therefore expand the scope of NLO organic materials.

## Data availability

Experimental details, synthesis and NMR spectra, stability measurements, and DFT simulation details. Crystallographic information files of TM1 (CCDC 2180022) and TM2 (CCDC 2180023).†

## Author contributions

R. Zhao synthesized the materials, grew the single crystals, carried out the structure characterization, and wrote the manuscript. T. Zhu did the DFT simulations. R. Zhao and S. Wang measured the SHG. C. J.-Wilkins, A. Najjarian, and S. Hoogland provided suggestions for the project. A. Lough resolved the single crystal structures. E. Sargent and D. Seferos directed this project. All the authors provided suggestions and comments on the manuscript.

## Conflicts of interest

There are no conflicts to declare.

## Supplementary Material

SC-013-D2SC03994J-s001

SC-013-D2SC03994J-s002
