# Supplementary material for: Engineering hydrogen bonding to align molecular dipoles in organic solids for efficient second harmonic generation
Source: Chem Sci. 2022 Oct 11;13(41):12144–8. doi: 10.1039/d2sc03994j (PMC9601317; doi:10.1039/d2sc03994j)
Supplement: SC-013-D2SC03994J-s001 [file SC-013-D2SC03994J-s001.pdf]

Electronic Supplementary Material (ESI) for Chemical Science.
This journal is © The Royal Society of Chemistry 2022

Electronic Supplementary Information (ESI)

Engineering Hydrogen Bonding to Align Molecular Dipoles in Organic Solids for Efficient Second Harmonic Generation

Ruyan Zhao,[†] Tong Zhu,[‡] Sasa Wang,[‡] Charlie Jarrett-Wilkins,[†] Amin Morteza Najjarian,[‡] Alan J. Lough,[†] Sjoerd Hoogland,[‡] Edward H. Sargent*[‡] and Dwight S. Seferos*[†⊥]

[†]*Department of Chemistry, University of Toronto*

80 St. George Street, Toronto, Ontario M5S 3H6, Canada

[‡]*Department of Electrical and Computer Engineering, University of Toronto,*

10 King's College Road, Toronto, Ontario M5S 3G4, Canada

[⊥]*Department of Chemical Engineering and Applied Chemistry, University of Toronto*

200 College Street, Toronto, Ontario M5S 3E5, Canada

E-mail: ted.sargent@utoronto.ca

dwight.seferos@utoronto.ca

Table of Contents

1. Experimental section
2. Synthesis of **TM1** and **TM2**
3. Material characterizations and crystal packing
4. DFT simulations
5. References

Summary of Figures

Fig. 1. FTIR spectra of **TM1** and **TM2**

Fig. S2. The transmittance of the powder sample of **TM1**

Fig. S3. TGA plot of **TM1**

Fig. S4. The π-π stacking distance in **TM1** and **TM2** crystals

Fig. S5. Molecular geometries and molecular orbitals of **7** and **TM1**

Fig. S6. Potential energy scan by changing dihedral angle between pyridine and thiophene

Fig. S7. SHG intensity of **TM1** *vs*. particle size at 1350 nm.

Fig. S8. Comparation between SHG intensity, band gap, and thermal stability

Fig. S9. DFT calculated total energy, band gap, and Berry phase polarizations

Fig. S10. Calculated band structure

Fig. S11. ^{1}H NMR spectrum of **3**

Fig. S12. ^{13}C NMR spectrum of **3**

Fig. S13. ^{1}H NMR spectrum of **4**

Fig. S14. ^{13}C NMR spectrum of **4**

Fig. S15. ^{1}H NMR spectrum of **6**

Fig. S16. ^{13}C NMR spectrum of **6**

Fig. S17. ^{1}H NMR spectrum of **7**

Fig. S18. ^{13}C NMR spectrum of **7**

Fig. S19. ^{1}H NMR spectrum of **TM1**

Fig. S20. ^{13}C NMR spectrum of **TM1**

Fig. S21. ^{1}H NMR spectrum of **TM2**

Fig. S22. ^{13}C NMR spectrum of **TM2**

Table S1. Summary of SHG intensity, band gap, and T_d comparison of representative organic SHG active materials

Table S2. Crystal data and structure refinement of **TM1**

Table S3. Hydrogen bonding in **TM1** crystal

Table S4. Crystal data and structure refinement of **TM2**

Table S5. Hydrogen bonding in **TM2** crystal

1. **Experimental Section**

Starting Materials. *Tert*-butyl ((5-bromothiophen-2-yl)methyl)carbamate 1, 3-fluoro-4-iodopyridine **1**, 3-fluoro-4-iodobenzonitrile **2**, (4-cyanophenyl)boronic acid **3**, thiophene-2-carbonitrile **4**, palladium(II)bis(triphenylphosphine) dichloridewere ($Pd(PPh_3)_2Cl_2$), CuI, TBAF, CF_3COOH, and DIPA were purchased from Sigma-Aldrich and used without any further purification.

Single crystal growth. Single crystals were grown using anti-solvent method. Firstly, the saturated solution of TM1 and TM2 was prepared in methanol, ether was utilized as the poor solvent. After evaporation overnight, crystals formed at the bottom of the vial.

Single crystal structure. Single-crystal structures were measured with Bruker Kappa APEX-DUO diffractometer equipped with a rotating anode with graphite-monochromated Mo-Kα radiation (Burker Triumph, λ = 0.71073 Å). The structures were solved by SHLEXT and SHELXL-2016/6, respectively.

Thermal stability. Thermal gravimetric analysis (TGA) of TM1 was carried out on a TA Instruments SDT Q600 thermogravimetric analyzer. TM1 powder was placed in an open platinum crucible, heating rate of 10 K/min from 30 ^{o}C to 800 ^{o}C under nitrogen atmosphere.

Linear absorption spectroscopy. Linear absorption spectra were collected using a PerkinElmerLAMBDA 950 UV/vis/NIR spectrophotometer with an integrating sphere.

pXRD patterns. pXRD patterns were collected on a Rigaku MiniFlex 600 6G Benchtop powder X-ray diffraction (XRD) instrument using Cu Kα radiation (λ= 1.5406 Å).

SHG measurements. The single crystal samples of TM1 and KDP were ground and sieved to make sure they have similar average particle size. Then the samples with the same particle size were pressed between two glass slides with an O ring interlayer with the diameter of 3 mm and thickness of 1 mm. The SHG measurements were carried out by the Kurtz-Perry method. A 1030 nm fundamental (5 kHz) was produced by an ytterbium-doped potassium gadolinium tungstate regenerative amplifier. The beam was then sent through an optical parametric amplifier (Orpheus, Light Conversion) to generate the wavelengths of 1350 nm. Scattered light from the thin films was then collected through two lenses into a 400 μm multimode fibre (Thorlabs), which was sent to a USB2000+ spectrometer (Ocean Optics).

DFT calculation details. Unless otherwise noted, all the calculations were performed using the FHI-aims all-electron code.[1–3] The default numerical settings, referred to as “intermediate” in FHI-aims were used. Local minimum-energy geometries of the Born-Oppenheimer surface were obtained with residual total energy gradients below 1×10^{-2} eV $Å^{-1}$ for atomic positions by PBE-GGA functional within the *vdW* correction following the Tkatcheko-Scheffler method (PBE+TS).[4,5] For the berry phase polarization calculations, the 2×2×1 supercell was used to explore the effect of the position of the fluorine atom. For all 16 different configurations, all the structures are fully relaxed by PBE+TS for the atomic positions and lattice constants by FHI-aims. Then the cp2k code is used to calculate the berry phase polarization based on these relaxed structures within PBE functional.[6] Goedecker-Teter-Hutter (GTH) pseudopotentials and the corresponding TZVP-GTH basis sets were employed.[7–9] The kinetic energy cutoff for the planewave expansions was set to 900 Ry. For the electrostatic potentials map, molecular dipole moment, and potential scan were performed on the basis set b3lyp/6-31G(d,p) using #opt=modredundant.

2. Synthesis of TM1 and TM2

Synthesis of *tert*-butyl ((5-((trimethylsilyl)ethynyl)thiophen-2-yl)methyl)carbamate 3. *Tert*-butyl ((5-bromothiophen-2-yl)methyl)carbamate **1** (1.000 g, 3.420 mmol), $Pd(PPh_3)_2Cl_2$ (72.00 mg, 0.103 mmol), and CuI (9.780 mg, 0.0.051 mmol) was added to a flame-dried flask containing 10 mL dry, and degassed THF. DIPA (865.2 mg, 1.210 mL, 8.550 mmol) and ethynyltrimethylsilane **2** (0.670 g, 0.940 mL, 6.840 mmol) was added to the mixture via syringe. The solution was stirred at room temperature overnight. The solution was poured into water and extracted with CH_2Cl_2, and the organic phase was washed three times with brine. The crude product was purified by column chromatography (hexane:ethyl acetate = 17:3) and collected as a pale-yellow solid (950 mg, 90%). ^1H NMR (400 MHz, $CDCl_3$), δ 7.05 (d, J = 3.7 Hz, 1H), 6.77 (d, J = 3.6 Hz, 1H), 4.87 (s, 1H), 4.41 (d, J = 6.0 Hz, 2H), 1.46 (s, 9H), 0.23 (s, 9H). ^{13}C NMR (126 MHz, $CDCl_3$), δ 155.63, 144.28, 132.66, 125.26, 122.82, 98.88, 97.66, 80.09, 39.79, 28.51, 0.14.

Synthesis of *tert*-butyl ((5-ethynylthiophen-2-yl)methyl)carbamate 4. ((5-((trimethylsilyl)ethynyl)thiophen-2-yl)methyl)carbamate **3** (1.200 g, 0.004 mol) was dissolved in 6 mL THF/H_2O (5:1) mixture solution. TBAF (1 M, 5 mL, 0.005 mol) was added dropwise and stirred for 1 hour. Water was added to the mixture and extracted with CH_2Cl_2 three times. The combined organic phase was dried over anhydrous $MgSO_4$ and the solvent was removed under reduced pressure. The crude product was purified by column chromatography (hexane:ethyl acetate = 4:1). The target product (925 mg, 98%) was collected as a brown oil. ^1H NMR (400 MHz, $CDCl_3$), δ 7.11 (d, J = 3.6 Hz, 1H), 6.80 (d, J = 3.6 Hz, 1H), 4.90 (s, 1H), 4.44–4.37 (m, 2H), 3.31 (s, 1H), 1.46 (s, 9H). ^{13}C NMR (126 MHz, $CDCl_3$), δ 155.52, 144.52, 133.01, 125.10, 121.42, 81.17, 80.00, 39.61, 28.39, 28.36.

***Synthesis of tert*-butyl ((5-((3-fluoropyridin-4-yl)ethynyl)thiophen-2-yl)methyl)carbamate 6.** 3-Fluoro-4-iodopyridine **5** (334.5 mg, 1.500 mmol), $Pd(PPh_3)_2Cl_2$ (31.60 mg, 0.045 mmol), and CuI (4.300 mg, 0.023 mmol) was added to a flame-dried flask containing 13 mL dry, and degassed THF. **4** (356.4 mg, 1.500 mmol) and DIPA (227.7 mg, 318.0 μL, 2.250 mmol) was added to the mixture via syringe. The reaction was stirred at room temperature overnight. The mixture was poured into water and extracted with CH_2Cl_2 for three times. Combined organic phase was washed with brine and dried over $MgSO_4$, and then filtered. The solvent was removed under reduced

pressure. The crude product was further purified by column chromatography (hexane:ethyl acetate from 9:1 to 2:1). The target product (416 mg, 83%) was collected as a yellow solid. ^{1}H NMR (400 MHz, $CDCl_3$), δ 8.50 (s, 1H), 8.39 (d, J = 5.0 Hz, 1H), 7.40–7.33 (m, 1H), 7.23 (d, J = 3.7 Hz, 1H), 6.88 (d, J = 3.9 Hz, 1H), 4.97 (s, 1H), 4.47 (d, J = 6.0 Hz, 2H), 1.47 (s, 9H). ^{13}C NMR (100 MHz, $CDCl_3$), δ 159.62, 157.01, 146.59, 145.45, 138.41, 133.79, 126.07, 125.52, 120.82, 119.65, 119.51, 92.41, 83.99, 39.71, 28.37.

Synthesis of (5-((3-fluoropyridin-4-yl)ethynyl)thiophen-2-yl)methanamine 7. CF_3COOH (3 mL) was added dropwise to the CH_2Cl_2 solution (3 mL) of *tert*-butyl ((5-((3-fluoropyridin-4-yl)ethynyl)thiophen-2-yl)methyl)carbamate (200.0 mg, 0.602 mmol) and stirred for 1.5 hours at room temperature. The mixture was cooled to 0 °C and saturated aqueous solution of $NaHCO_3$ was added to the solution to tune the PH value to 10. The solution was extracted with CH_2Cl_2. The combined organic phase was dried over $MgSO_4$ and then filtered. The solvent was removed in vacuo and the target product (120 mg, 86%) was collected as a yellow solid. ^{1}H NMR (400 MHz, $CDCl_3$), δ 8.50 (d, J = 1.4 Hz, 1H), 8.39 (dd, J = 4.9, 1.0 Hz, 1H), 7.37 (ddd, J = 6.0, 4.9, 0.6 Hz, 1H), 7.25 (d, J = 3.7 Hz, 1H), 6.86 (dt, J = 3.6, 1.0 Hz, 1H), 4.07 (d, J = 1.0 Hz, 2H), 1.58 (s, 2H). ^{13}C NMR (101 MHz, $CDCl_3$), δ 159.61, 157.02, 152.07, 145.44, 138.38, 133.98, 126.06, 123.84, 119.86, 92.80, 83.79, 41.64.

Synthesis of TM1. **7** (300 mg, 1.293 mmol) was dissolved in methanol, 1 equivalent of dilute HBr in methanol was added dropwise to the solution to form the white precipitates. Ether was added before a large amount of solid was collected by filtration and washed with ether. The solvent was removed in vacuo and the target product (373 mg, 92%) was collected as a white powder. ^{1}H NMR (500 MHz, DMSO), δ 8.72 (d, J = 1.3 Hz, 1H), 8.50 (dd, J = 4.9, 1.1 Hz, 1H), 8.30 (s, 3H), 7.67 (dd, J = 6.2, 4.9 Hz, 1H), 7.54 (d, J = 3.7 Hz, 1H), 7.29 (dd, J = 3.7, 0.8 Hz, 1H), 4.31 (d, J = 0.8 Hz, 2H). ^{13}C NMR (126 MHz, DMSO), δ 158.68, 156.61, 146.19, 139.86, 138.32-132.09, 134.62, 130.06, 126.34, 120.98, 117.98, 91.42, 84.24, 36.75.

Synthesis of TM2. **7** (311.1 mg, 0.134 mmol) was dissolved in methanol, and 1 M HBr (0.4 mL, 0.4 mmol) was added dropwise to the solution until the yellow precipitates formed. Ether was added before a large amount of solid was collected by filtration and washed with ether. The solvent was removed in vacuo and the target product (475 mg, 90%) was collected as a yellow powder. ^{1}H NMR (400 MHz, DMSO), δ 8.73 (d, J = 1.5 Hz, 1H), 8.51 (dd, J = 4.9, 1.1 Hz, 1H), 8.31 (s,

3H), 7.69 (dd, *J* = 6.2, 4.9 Hz, 1H), 7.54 (d, *J* = 3.7 Hz, 1H), 7.29 (d, *J* = 3.8 Hz, 1H), 4.31 (d, J = 5.7 Hz, 2H). ^{13}C NMR (126 MHz, DMSO), δ 158.72, 156.66, 145.93, 139.94, 138.09, 134.73, 130.02, 126.46, 120.94, 118.34, 91.65, 84.24, 36.78.

3. Material Characterizations and Crystal Packing

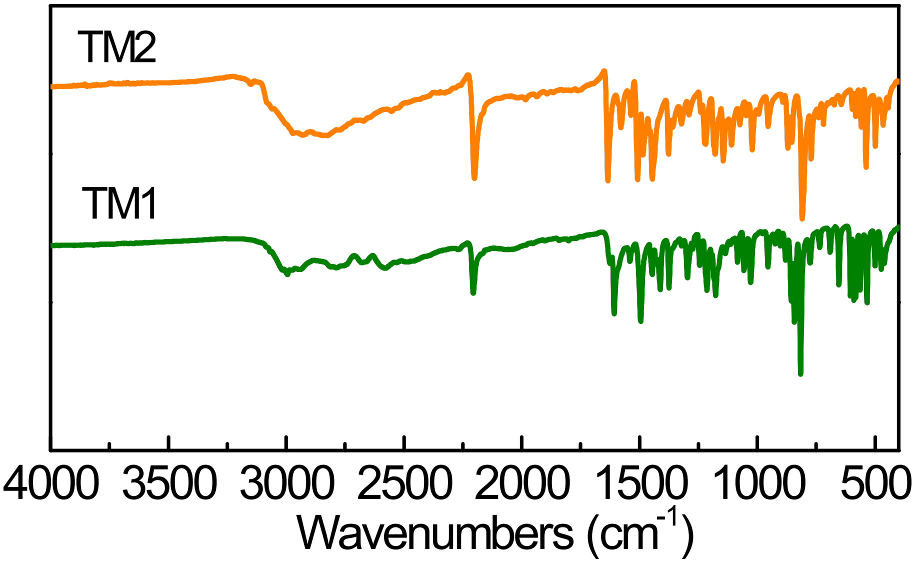

Fig. S1. FTIR spectra of **TM1** and **TM2**.

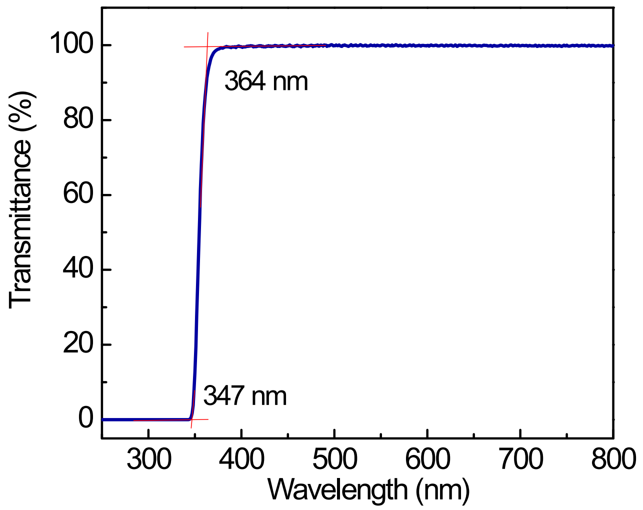

Fig. S2. Transmittance of the powder sample of **TM1**.

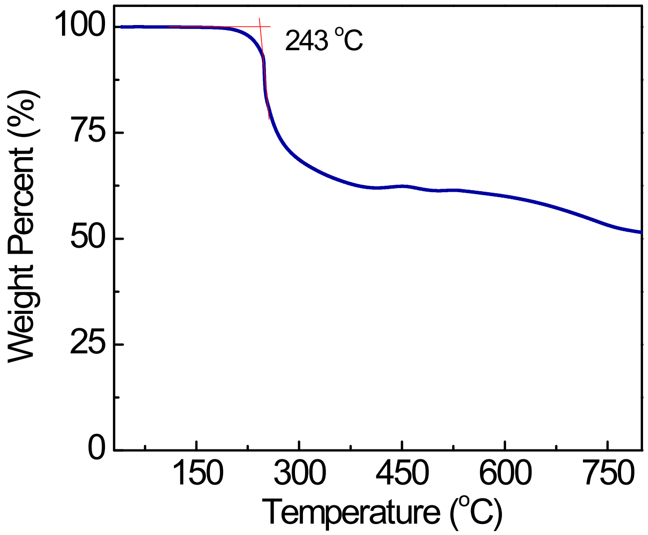

Fig. S3. TGA plot of **TM1**.

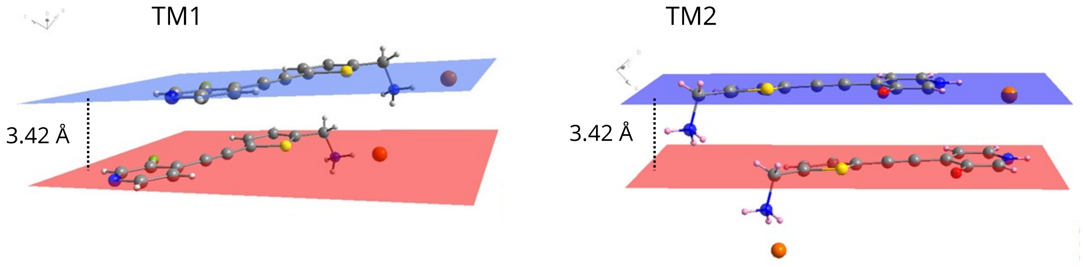

Fig. S4. π-π stacking distance in **TM1** and **TM2** crystals.

4. DFT Simulations

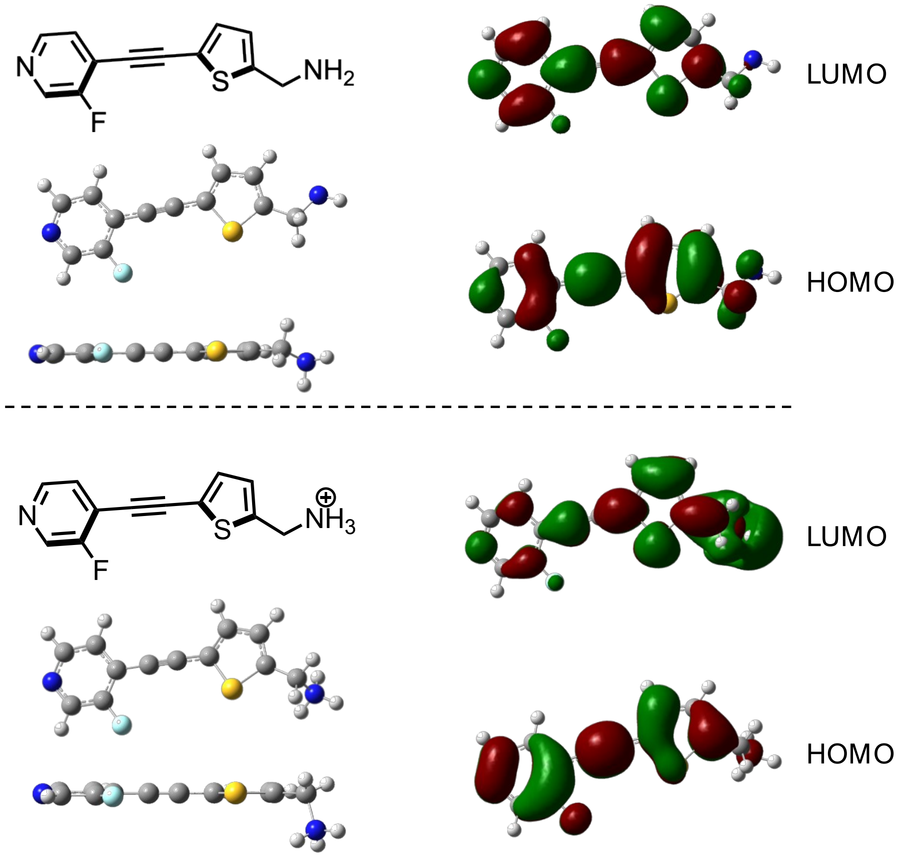

Fig. S5. Molecular geometries and molecular orbitals of neutralized and protonated **TM1**.

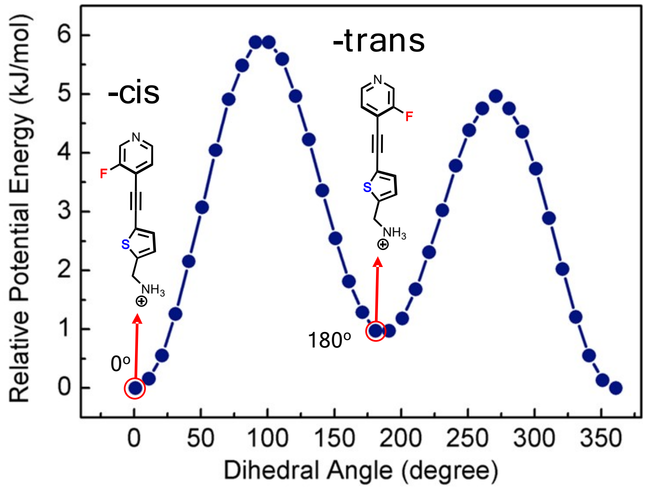

Fig. S6. Potential energy scan by changing the dihedral angle between pyridine and thiophene.

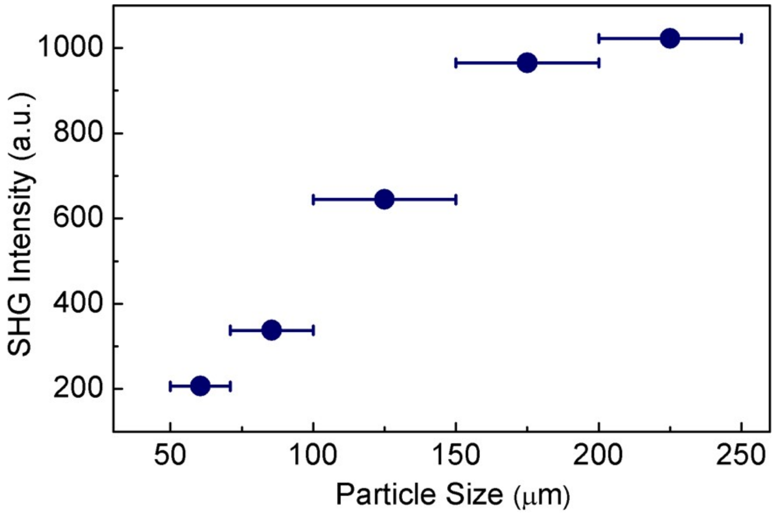

Fig. S7. SHG intensity of **TM1** *vs*. particle size at 1350 nm.

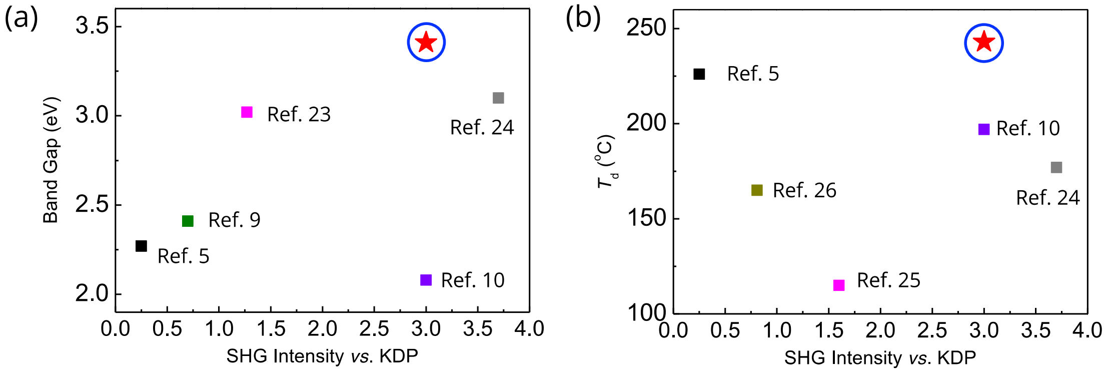

Fig. S8. (a,b) SHG intensity, band gap, and T_d comparison of representative organic SHG active materials (square: data from references, for clarity, the Reference numbers are in accordance with the main text; circled red star: this work).

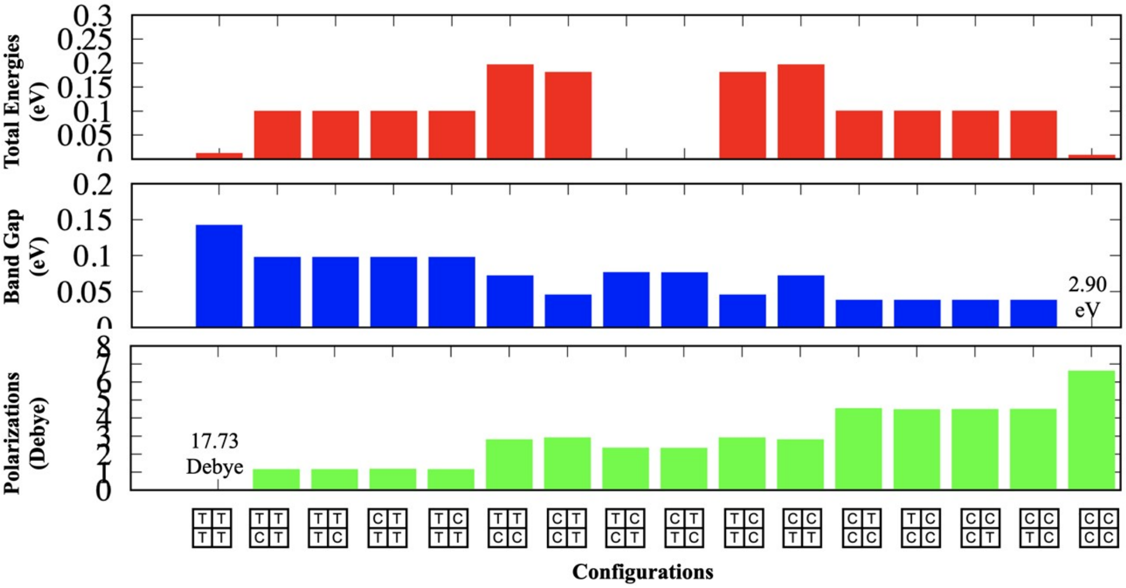

Fig. S9. DFT calculated Total energies (in eV), Band gaps (in eV), and Berry phase polarizations (in Debye) for all the 16 different configurations in 2 × 2 × 1 supercell. The C stands for the -cis conformations and T stands for the -trans conformations.

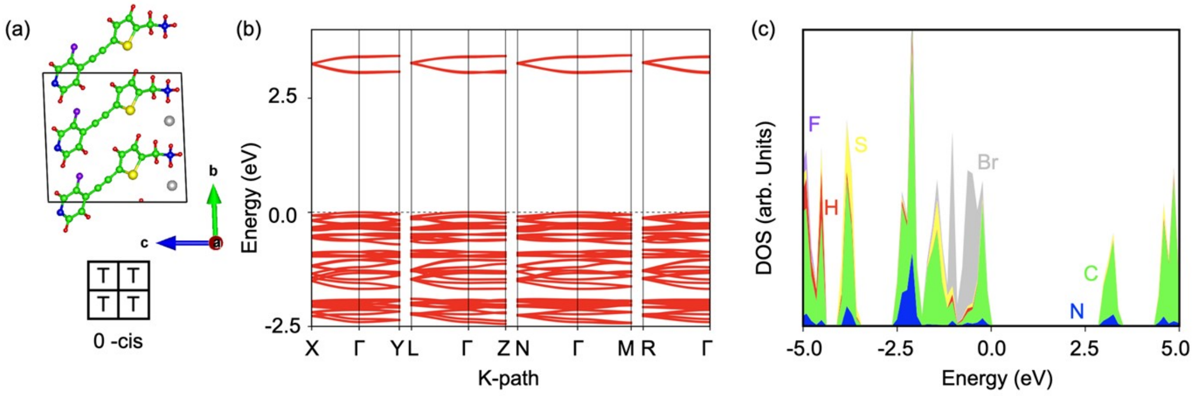

Fig. S10. (a) 0-cis alignments (b) Calculated band structure of **TM1**. (c) Total and elemental-decomposed density of states (DOS).

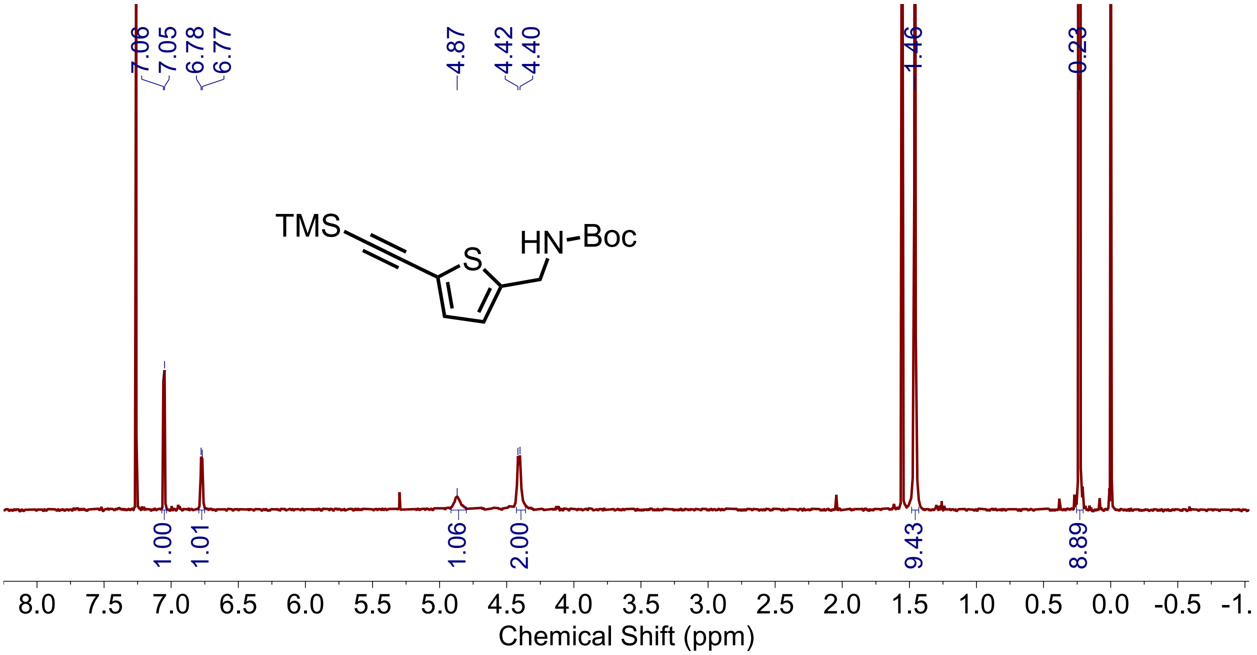

Fig. S11. ^{1}H NMR (400 MHz, $CDCl_3$, 25 ^{o}C) spectrum of *tert*-butyl ((5-((trimethylsilyl)ethynyl)thiophen-2-yl)methyl)carbamate **3**.

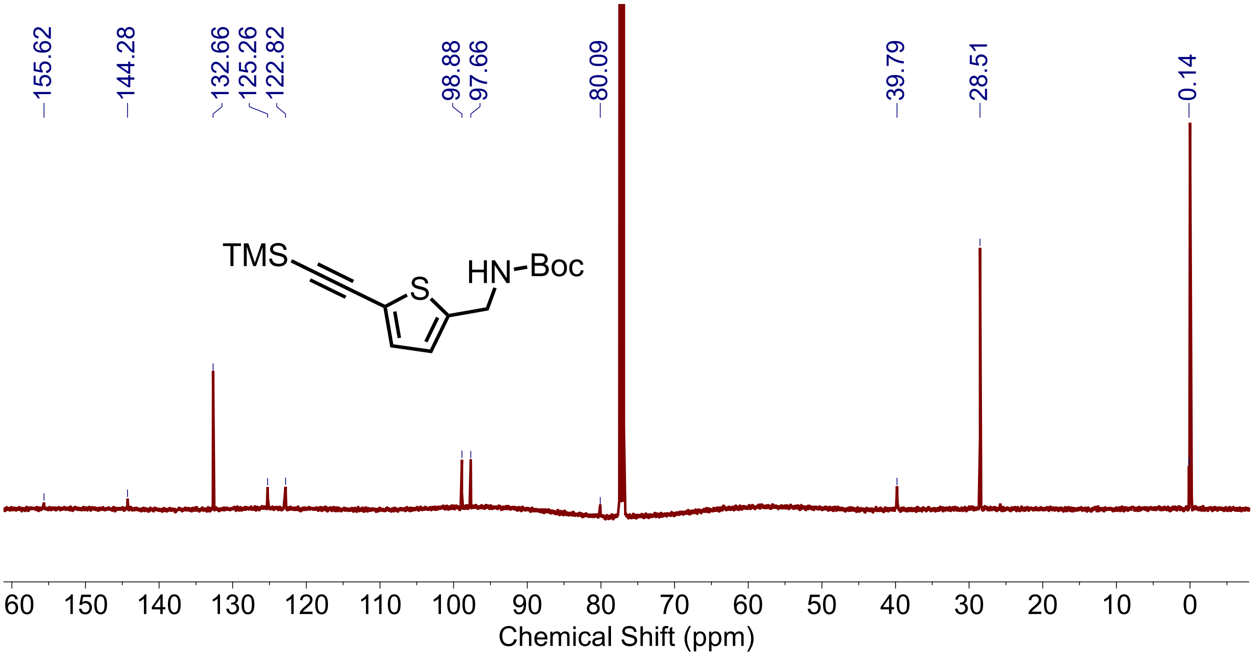

Fig. S12. ^{13}C NMR (126 MHz, $CDCl_3$, 25 ^{o}C) spectrum of *tert*-butyl ((5-((trimethylsilyl)ethynyl)thiophen-2-yl)methyl)carbamate **3**.

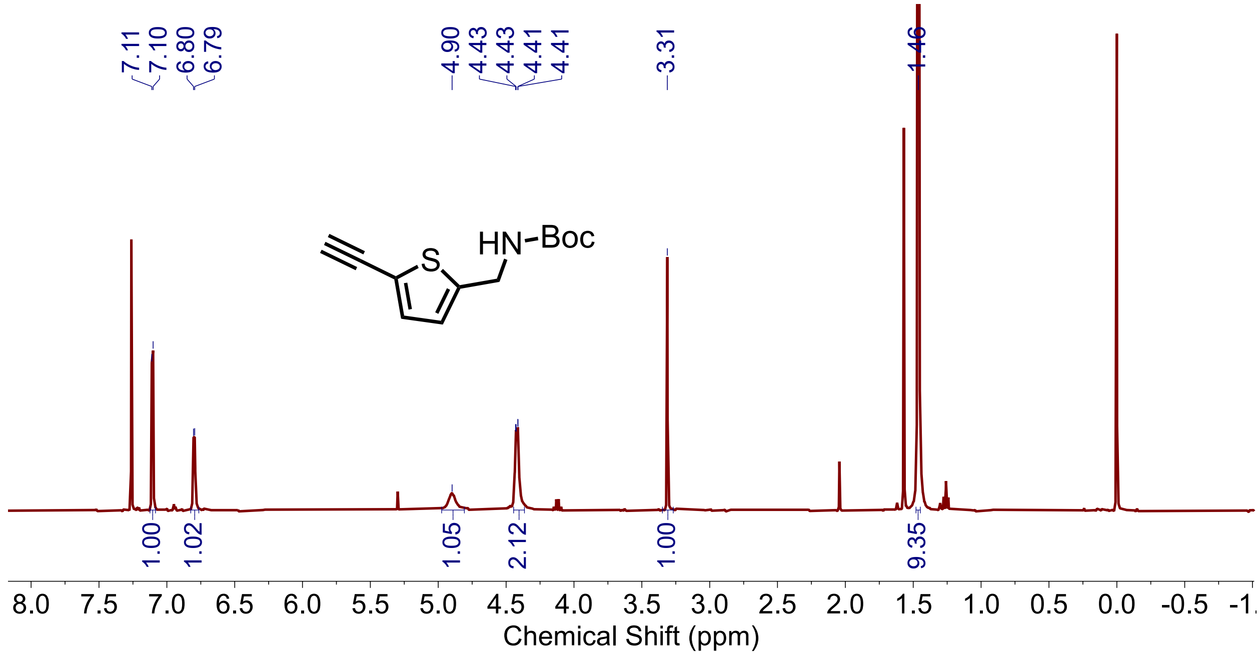

Fig. S13. ^{1}H NMR (400 MHz, $CDCl_3$, 25 ^{o}C) spectrum of *tert*-butyl ((5-ethynylthiophen-2-yl)methyl)carbamate **4**.

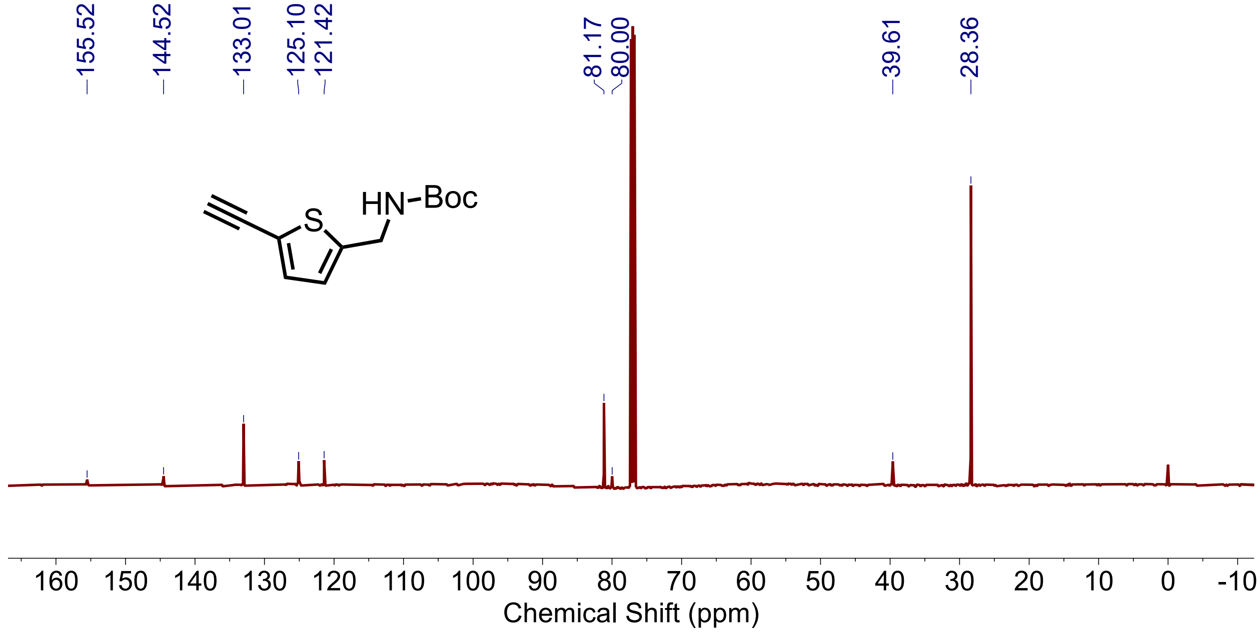

Fig. S14. ^{13}C NMR (126 MHz, $CDCl_3$, 25 ^{o}C) spectrum of *tert*-butyl ((5-ethynylthiophen-2-yl)methyl)carbamate **4**.

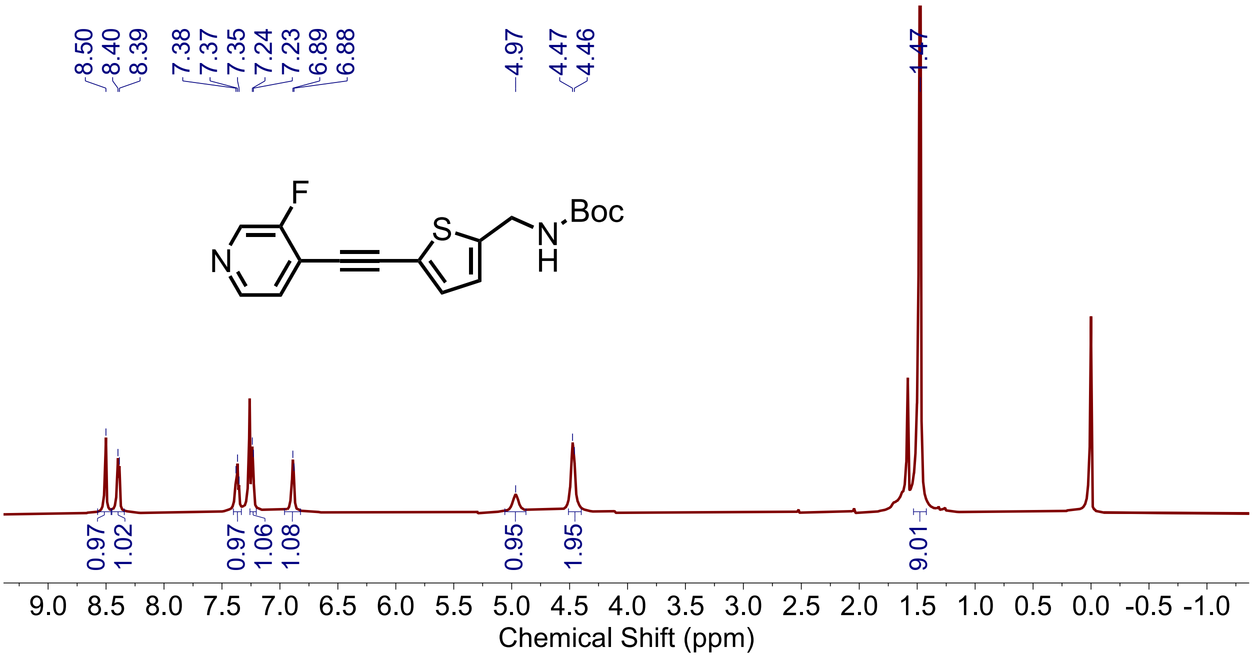

Fig. S15. ^{1}H NMR (400 MHz, $CDCl_3$, 25 °C) spectrum of *tert*-butyl ((5-((3-fluoropyridin-4-yl)ethynyl)thiophen-2-yl)methyl)carbamate **6**.

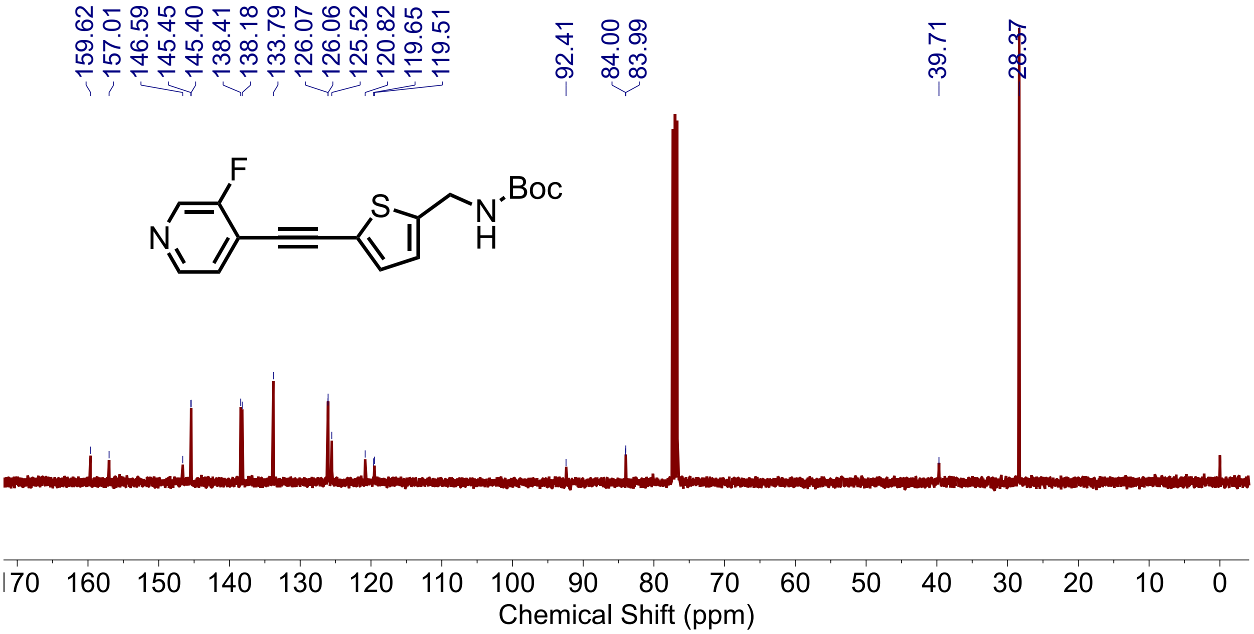

Fig. S16. ^{13}C NMR (101 MHz, $CDCl_3$, 25 °C) spectrum of *tert*-butyl ((5-((3-fluoropyridin-4-yl)ethynyl)thiophen-2-yl)methyl)carbamate **6**.

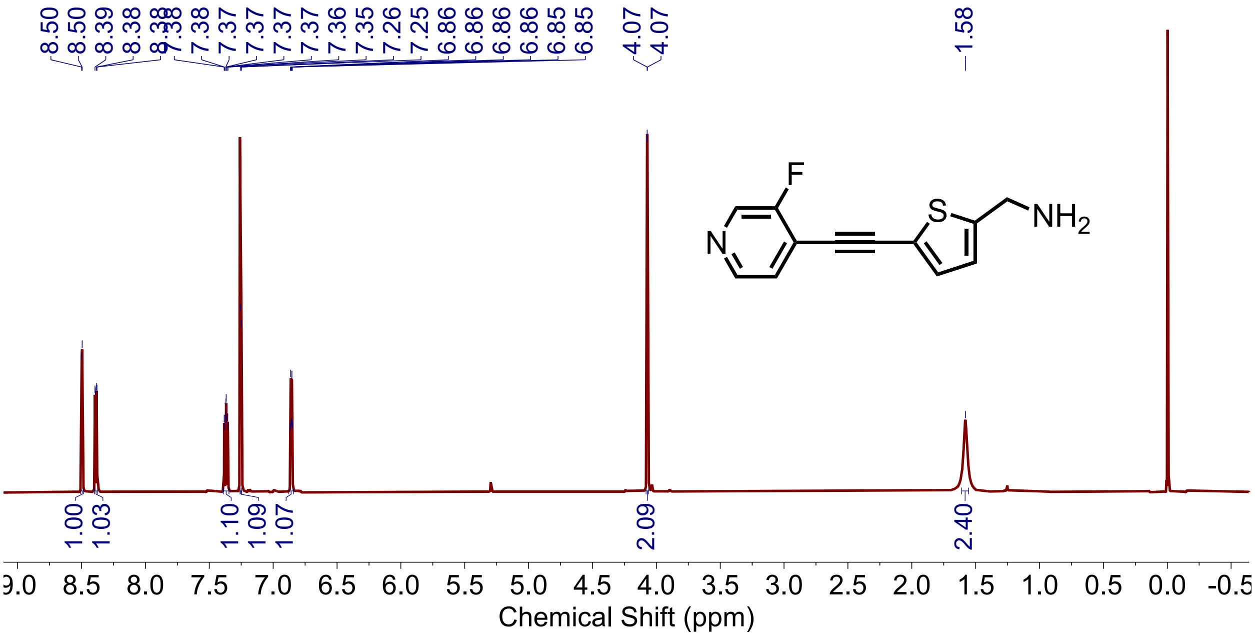

Fig. S17. ^{1}H NMR (400 MHz, $CDCl_3$, 25 °C) spectrum of (5-((3-fluoropyridin-4-yl)ethynyl)thiophen-2-yl)methanamine **7**.

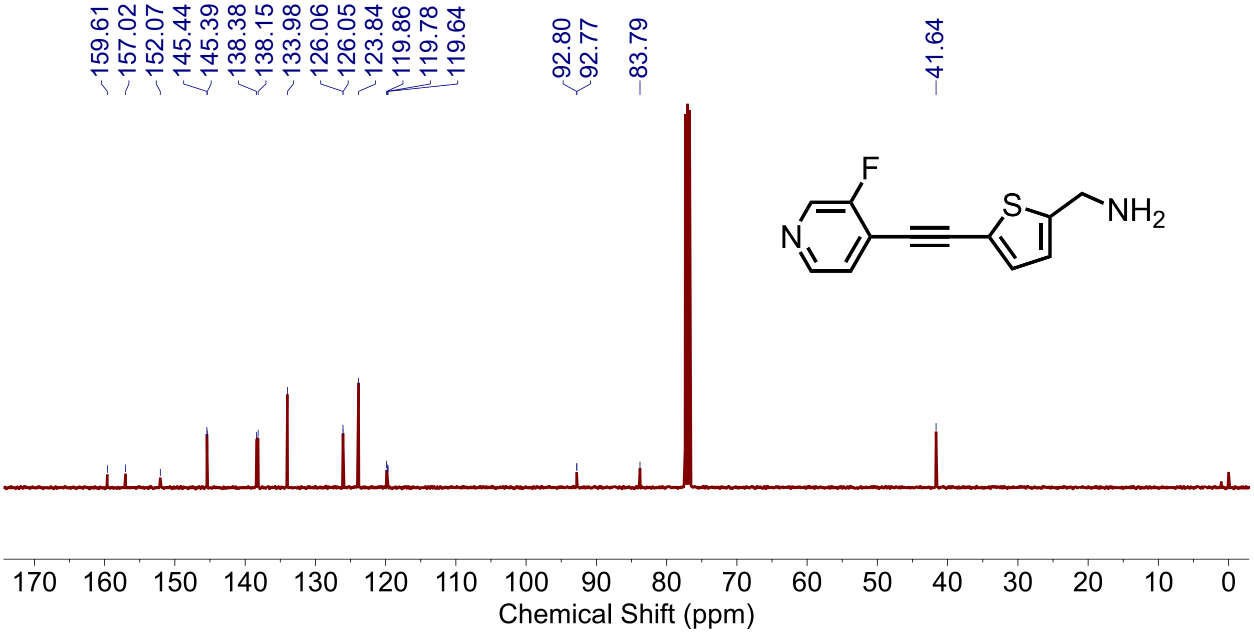

Fig. S18. ^{13}C NMR (101 MHz, $CDCl_3$, 25 °C) spectrum of (5-((3-fluoropyridin-4-yl)ethynyl)thiophen-2-yl)methanamine **7**.

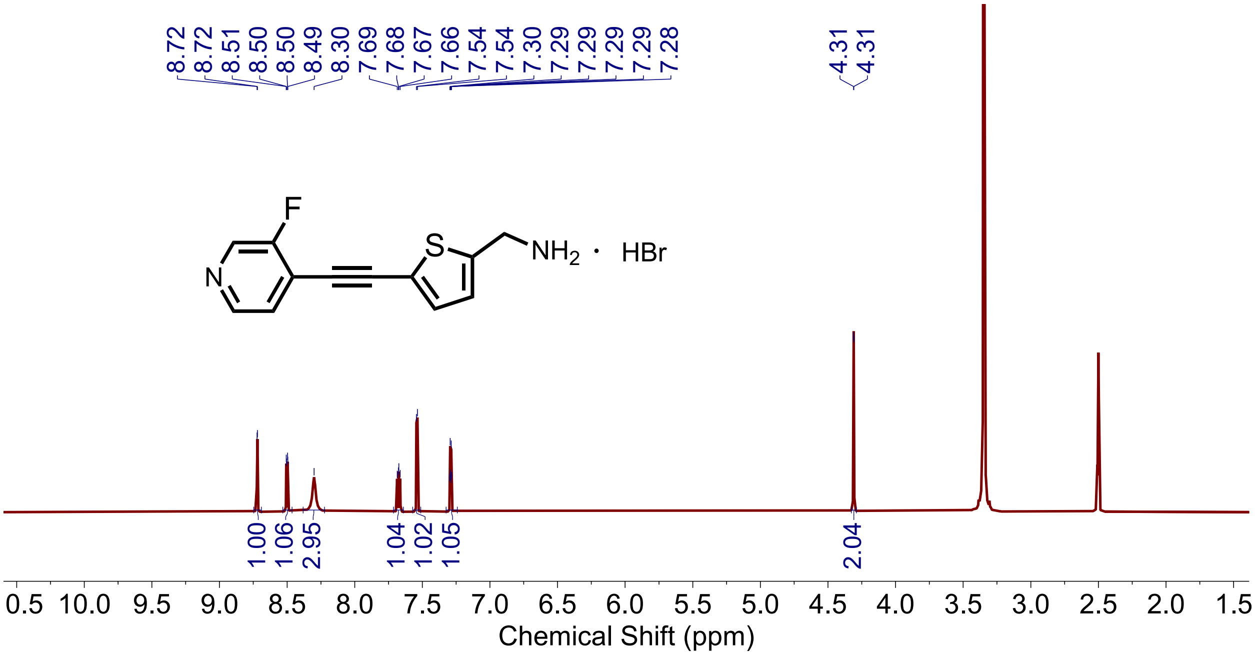

Fig. S19. ^{1}H NMR (500 MHz, DMSO, 25 ^{o}C) spectrum of **TM1**.

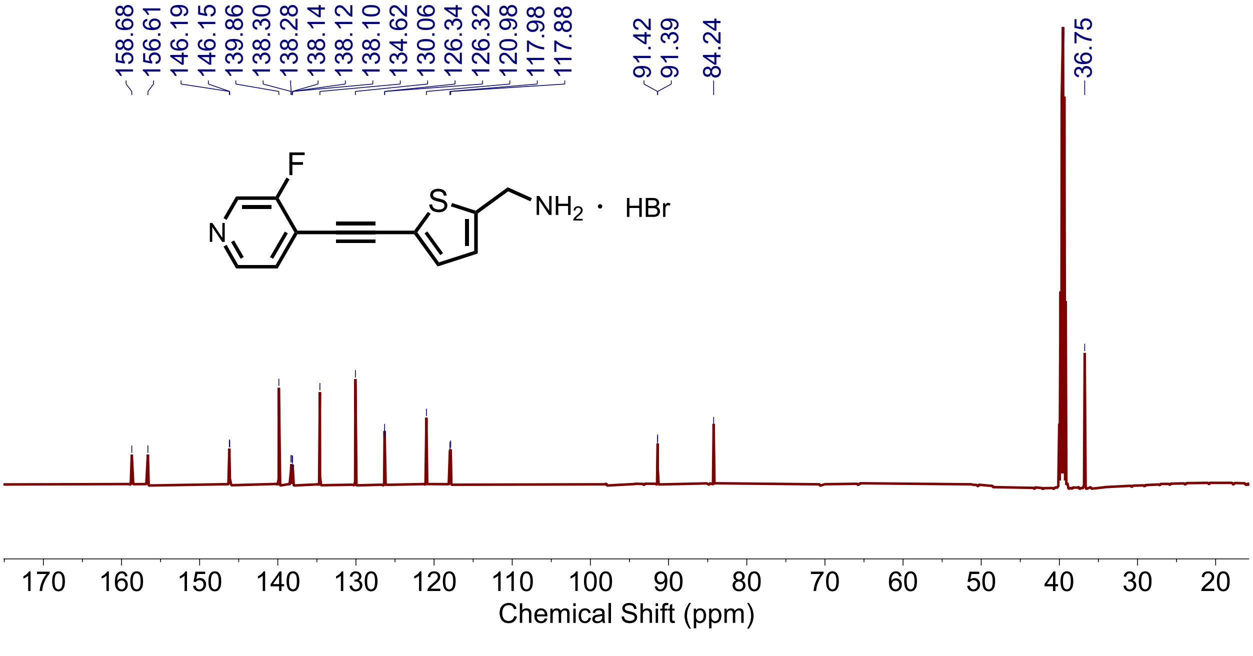

Fig. S20. ^{13}C NMR (126 MHz, DMSO, 25 ^{o}C) spectrum of **TM1**.

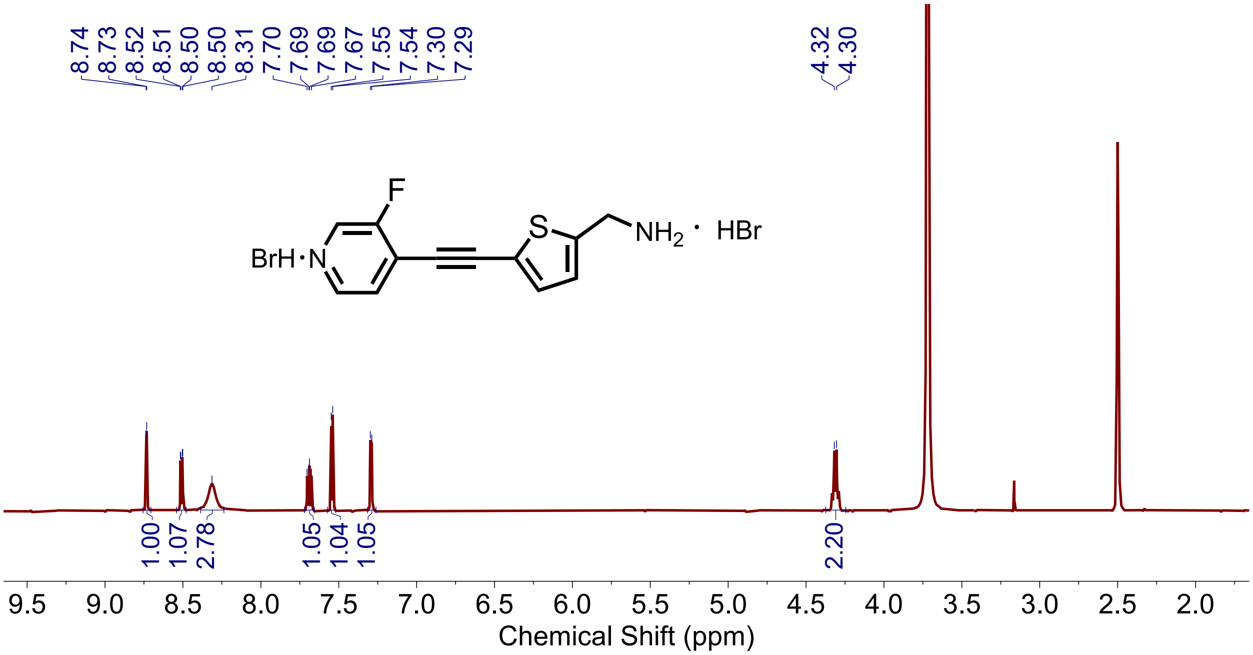

Fig. S21. ^{1}H NMR (400 MHz, DMSO, 25 °C) spectrum of **TM2**.

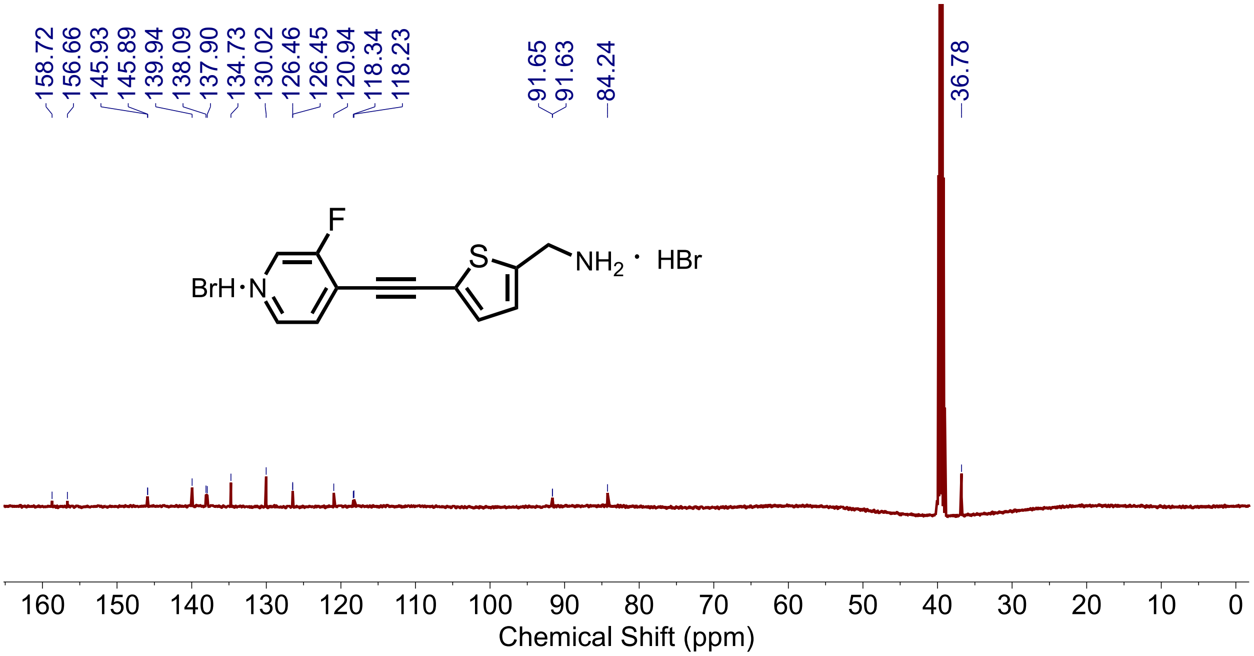

Fig. S22. ^{13}C NMR (126 MHz, DMSO, 25 °C) spectrum of **TM2**.

Table S1. Summary of SHG intensity, band gap, and T_d comparison of representative organic SHG active materials.

Chemical structure	SHG intensity (*vs*. KDP)	Band gap (eV)	T_d (°C)	Reference
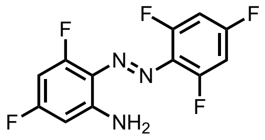	0.25	2.27	226	5
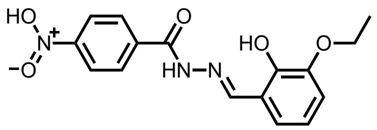	0.70	2.41	/	9
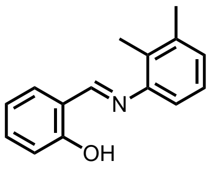	3	2.08	197	10
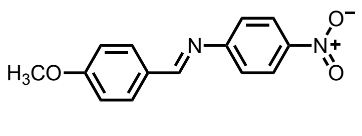	1.27	3.02	/	21
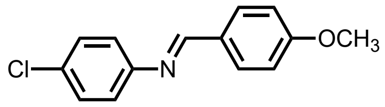	3.7	3.17	177	22

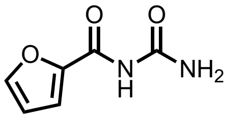	1.6	/	120	23
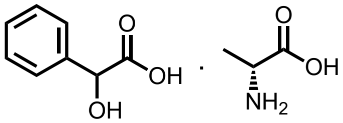	0.81	/	165	24
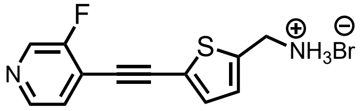	3	3.57	243	*this work*

Table S2. Crystal data and structure refinement for **TM1**.

Name	**TM1**
Empirical formula	C12 H10 Br F N2 S
Formula weight	313.19
Temperature	150(2) K
Wavelength	0.71073 Å
Crystal system	Triclinic
Space group	P1
Unit cell dimensions	a = 4.5926(3) Å, α = 86.311(2)°<br>b = 5.6626(4) Å, β = 88.909(2)°<br>c = 12.1650(8) Å, γ = 81.003(2)°
Volume	311.81(4) $Å^3$
Z	1
Density (calculated)	1.668 Mg/m^3
Absorption coefficient	3.453 mm^{-1}
F(000)	156
Crystal size	0.190 x 0.160 x 0.070 mm^3
Theta range for data collection	1.678 to 27.602°
Index ranges	-5 <= h <= 5, -7 <= k <= 7, -15 <= l <= 15
Reflections collected	4945
Independent reflections	2260 [R(int) = 0.0276]
Completeness to theta = 25.242°	99.8%
Absorption correction	Semi-empirical from equivalents
Max. and min. transmission	0.7456 and 0.6118
Refinement method	Full-matrix least-squares on F^2
Data / restraints / parameters	2260 / 3[a] / 161
Goodness-of-fit on F^2	1.053
Final R indices [I > 2sigma(I)]	R1 = 0.0228, wR2 = 0.0332
R indices (all data)	R1 = 0.0249, wR2 = 0.0334
Absolute structure parameter	0.371(7)
Extinction coefficient	n/a
Largest diff. peak and hole	0.322 and -0.282 e.$Å^{-3}$

[a]The fluorine atoms of **TM1** are disordered over the two ortho sites of the benzene ring with refined occupancies of 0.836(5) and 0.164(5). A model was set up to refine this disorder where the carbon atoms of the ring were split accordingly to match the occupancies of the F atoms. This action required restraints to be applied to the carbon atoms in the SHELXL software in the form of EXYZ C9 C9A, EADP C9 C9A, EXYZ C12 C12A, and EADP C12 C12A.

Table S3. Hydrogen bonds for **TM1** [Å and °].

D-H...A	d (D-H)	d (H...A)	d (D...A)	angle (DHA)
N(1)-H(1C)...N(2)#1	0.91	1.97	2.860(4)	165.2
N(1)-H(1D)...Br(1)#2	0.91	2.49	3.305(3)	149.0
N(1)-H(1E)...Br(1)#3	0.91	2.53	3.386(2)	157.1

Symmetry transformations used to generate equivalent atoms:

#1 x+1,y+1,z-1   #2 x-1,y+1,z   #3 x-1,y,z

Table S4. Crystal data and structure refinement for **TM2**.

Name	**TM2**
Empirical formula	C12 H11 Br2 F N2 S
Formula weight	394.11
Temperature	150(2) K
Wavelength	0.71073 Å
Crystal system	Triclinic
Space group	P-1
Unit cell dimensions	a = 4.5711(10) Å, α = 69.971(6)° b = 12.769(3) Å, β = 89.227(6)° c = 13.073(3) Å, γ = 85.826(6)°
Volume	714.9(3) $Å^3$
Z	2
Density (calculated)	1.831 Mg/m^3
Absorption coefficient	5.810 mm^{-1}
F(000)	384
Crystal size	0.180 x 0.120 x 0.040 mm^3
Theta range for data collection	1.658 to 27.478°
Index ranges	-5 <= h <= 5, -16 <= k <= 16, -16 <= l <= 16
Reflections collected	16185
Independent reflections	3253 [R(int) = 0.0615]
Completeness to theta = 25.242°	100.0%
Absorption correction	Semi-empirical from equivalents
Max. and min. transmission	0.7455 and 0.6228
Refinement method	Full-matrix least-squares on F^2
Data / restraints / parameters	3253 / 0 / 179
Goodness-of-fit on F^2	1.010
Final R indices [I > 2sigma(I)]	R1 = 0.0309, wR2 = 0.0539
R indices (all data)	R1 = 0.0563, wR2 = 0.0581
Extinction coefficient	n/a
Largest diff. peak and hole	0.464 and -0.534 e.$Å^{-3}$

Table S5. Hydrogen bonds for **TM2** [Å and °].

D-H...A	d(D-H)	d(H...A)	d(D...A)	<(DHA)
N(1)-H(1N)...Br(1)	0.93(4)	2.41(4)	3.321(3)	168(3)
N(1)-H(2N)...Br(2)#1	0.97(3)	2.41(3)	3.382(3)	174(3)
N(1)-H(3N)...Br(1)#2	0.93(3)	2.51(3)	3.332(3)	148(2)
N(2)-H(4N)...Br(2)	0.88(3)	2.49(3)	3.263(3)	147(3)

Symmetry transformations used to generate equivalent atoms:

#1 x+1,y+1,z   #2 -x+2,-y+2,-z+1

5. References

1. Blum, V. et. al. Comput. Phys. Commun. 2009, 180, 2175-2196.
2. Havu, V. et. al. J. Comput. Phys. 2009, 228, 8367-8379.
3. Ren, X. et. al., New J. Phys. 2010, 14, 053020.
4. Perdew, J. P., et. al., Phys. Rev. Lett. 77, 3865. (1996) Erratum Phys. Rev. Lett. 78, 1396 (1997)
5. Tkatchenko, A., et. al., Phys. Rev. Lett. 2009, 102, 073005.
6. CP2K Developers Group, URL: http://www.cp2k.org.
7. S. Goedecker, M. Teter, and J. Hutter, Phys. Rev. B, 1996, 54, 1703.
8. Hartwigsen, S. Goedecker, and J. Hutter, Phys. Rev. B 1998, 58, 3641.
9. Krack, Theor. Chem. Acc. 2005, 114, 145.
